# Magnetite sand as a low-cost material for electromagnetic shielding and mechanical enhancement of concrete

**DOI:** 10.1038/s41598-026-47469-8

**Published:** 2026-05-07

**Authors:** Shady H. El-Gohary, Wegdan W. El-Nadoury, Ehab A. Kholief, Ahmed S. I.Amar, Adel M. Soliman

**Affiliations:** 1 Faculty of Artificial Intelligence, Delta University for Science and Technology, Gamasa 35712, Egypt; 2 Department of Communication, Air Defense College, Egyptian Military Academy, New Capital 4804004, Egypt; 3 Civil Engineering Department, Faculty of Engineering, Horus University-Egypt, New Damietta, Egypt; 4 Industrial Engineering, Egypt Black Sand Company, Cairo, Egypt; 5 Electronic Communication Department, Egyptian Technical Research and Development Centre, Cairo, Egypt; 6 Electronics and Communications Department, Faculty of Engineering, Horus University-Egypt, New Damietta, Egypt

**Keywords:** Electromagnetic, Shielding, Concrete, Magnetite, Engineering, Materials science

## Abstract

The widespread use of electromagnetic waves in various fields, such as communications, medicine, and household use, has become a tangible reality. However, this increasing use is surrounded by concerns about the harmful effects of electromagnetic waves. In this study, samples of concrete used in buildings were prepared, and conventional sand was replaced with magnetite sand extracted from Egyptian black sand at ratios of 10, 20, 30, and 40% of the total sand content. Their ability to absorb electromagnetic waves in the frequency range of 2 to 12 GHz was evaluated. The results showed that magnetite can absorb electromagnetic waves with an efficiency of up to 18 dB at a frequency of 8 GHz for the sample in which conventional sand was replaced by magnetite sand by 40%. Moreover, the addition of magnetite sand to the concrete improved the mechanical properties. The compressive strength increased progressively from 35 to 47.25 MPa, and the tensile strength improved from 4.14 to 4.80 MPa. There were also less pronounced cracks, more compact fractured surfaces, and signs of energy dissipation, indicating quasi-brittle or tougher behavior.

## Introduction

The unique advantages of electromagnetic waves (EMW) in terms of cost and efficiency, extensive coverage, and adaptability to challenging environments have solidified their indispensable role in the current and future perspectives of telecommunications, such as radars, mobile phones, radio and television transmitters, communication towers, satellites, microwave ovens, and some devices used in medical and industrial practice. As technological demands continue to escalate, further research into optimizing EMW utilization and enhancing spectral efficiency is needed. This has raised questions in the minds of scientists about the impact of the increasing use of electromagnetic waves on health. Unfortunately, this significant progress causes intense electromagnetic pollution, both in terms of the duration of exposure to these waves and their wave strength, on living organisms and human health^1^. Some studies have linked maternal exposure to electromagnetic waves during pregnancy to adverse fetal complications, such as a significant increase in oxidative factors, a decrease in antioxidant factors, an increase in DNA damage parameters, and changes in protein expression in umbilical cord blood genes^2,3^. Furthermore, there are suspicions that EMW affects the vital functions of the heart^4,5^. Therefore, intensive efforts have recently been made to eliminate electromagnetic pollution and reduce its impact on human life by reducing reliance on electromagnetic waves or by blocking them. One such solution is to use buildings that are impermeable to electromagnetic waves by incorporating materials that reflect or absorb electromagnetic waves into the building materials, thereby isolating or reducing the impact of electromagnetic waves on those inside the buildings. In addition to another application, it is to prevent electromagnetic waves from penetrating the buildings to maintain the privacy of information within a single room^6–8^.

### Methodology

**Fig. 1:**
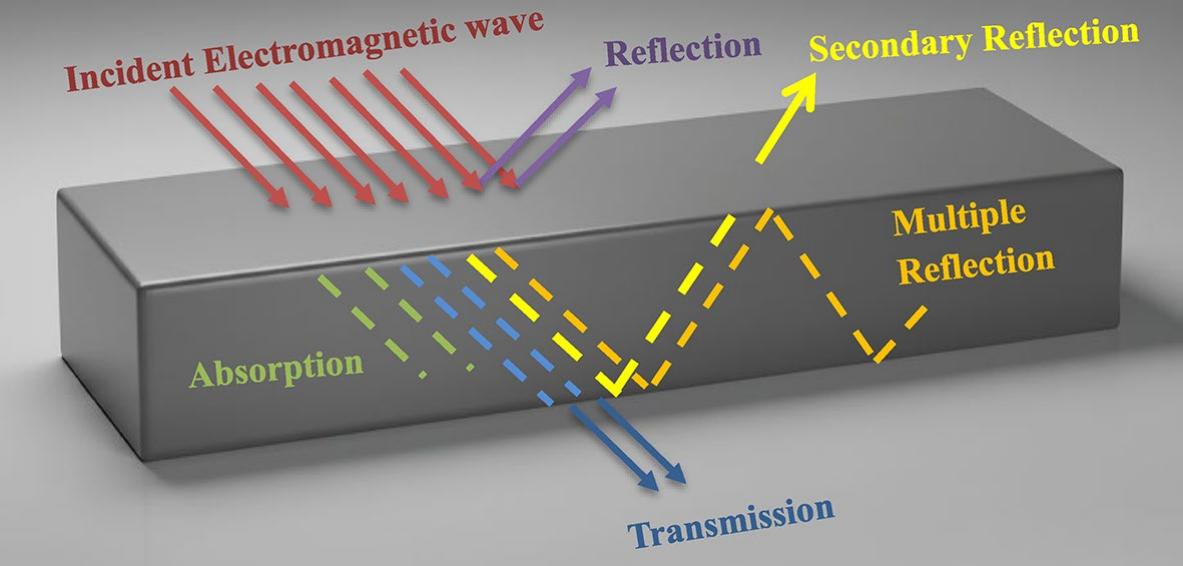
Schematic representation of microwave interaction with matter.

Electromagnetic shielding effectiveness (EMSE) measures how well a material or structure reduces the intensity of an electromagnetic field that passes through it. So, it quantifies the attenuation of electromagnetic radiation provided by a shield (Fig. 1).

The EMSE is typically expressed in decibels (dB) and represents the ratio of the electromagnetic power (or field strength) before and after the insertion of the shield. Mathematically, EMSE in dB is defined as:

1 $${EMSE}_{dB}=10\mathrm{log}\frac{{P}_{i}}{{P}_{t}}=20\mathrm{log}\frac{{E}_{i}}{{E}_{t}}=20\mathrm{log}\frac{{H}_{i}}{{H}_{t}}$$

where ${P}_{i}$, ${P}_{t},$ *E*_*i*_*, E*_*t*_*, H*_*i*_*,* and *H*_*t*_ are the power of the electromagnetic wave incident on the shield, the power of the electromagnetic wave transmitted through the shield, the electrical field strength, the transmitted electrical field strength, the incident magnetic field strength, transmitted magnetic field strength, respectively. A higher positive dB value indicates better shielding performance, meaning a greater reduction in the electromagnetic field strength. When evaluating the EMSE of a material using a Vector Network Analyzer (VNA), the forward transmission coefficient S21 parameter is the most relevant. The magnitude of S21 (∣S21∣) represents the ratio of the power transmitted through the material to the power incident on it. To determine the EMSE in dB from the S21 parameter, the transmission with and without the shielding material in place is compared. The measurement of the S21 parameter without the shielding material (S21_ref_) establishes a baseline for the transmission through the system. The measurement of the S21 parameter with the shielding material (S21_shield_) is measured under the same conditions. The value of (S21_shield_) represents the attenuation of electromagnetic waves when transmitted through the system with the shielding material. The EMSE in dB can be calculated as the difference between the magnitude of S21 measured without and with the shield, expressed in dB^9,10^.

2 $${EMSE}_{dB}={S21}_{ref}-{S21}_{shield}$$

### Cement-based materials for EMW shielding

Cement-based materials can be modified by the addition of some conductive and /or dielectric, and/or magnetic materials to enhance the electromagnetic shielding ability of the matrix material^11^. Therefore, some studies have adopted the addition of materials such as Steel microfiber, carbon fiber, Fly ash, iron oxide, carbon nanofiber, carbon fiber, carbon black nanoparticles, graphene nanoplatelets, waste copper swarf, Zinc oxide^12–14^, ferrite particles, manganese zinc ferrite (Mn–Zn ferrite), nano magnetite (Fe_3_O_4),_ expanded graphite. The evaluation centers on these materials’ effectiveness in isolating internal electromagnetic waves, considering both their cost and influence on building mechanical properties^15–19^.

## Materials and methods

### Materials

**Fig. 2:**
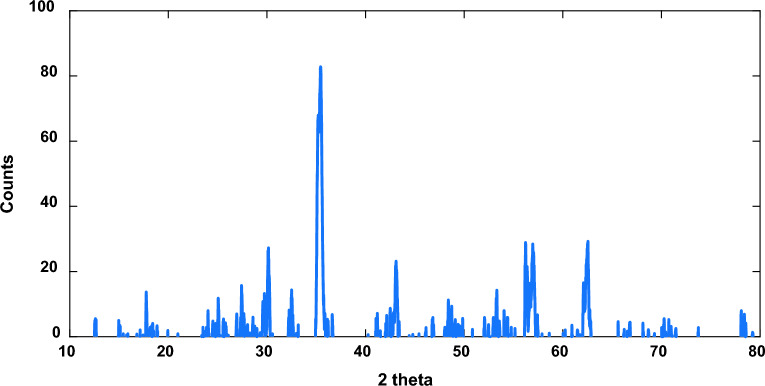
XRD of the magnetite sample.

Magnetite extracted from the Egyptian Black Sand Company. The Magnetite XRD characterization, as shown in Fig. 2, showed that magnetite Fe_3_O_4_ characteristic peaks can be recognized at 2θ positions = 18.01° (111), 30.284° (220), 35.662° (311), 43.289° (400), 53.5° (422), 57.173° (511), 62.844° (440)^20^. Where Titanium dioxide TiO_2_. peaks were recognized at 2θ positions = 27.42° (110), 36.08°(101), 41.25° (111), 54.33° (211) and 63.44° (002) Silicon dioxide SiO_2_^21,22^. Peaks appeared at 2θ = 28.38°(111), 47.26°(220), and 56.08°(311). Peaks at 2θ = 36.862° (111), 42,824° (002), 62.167° (022), 74.716° (113), and 78.443° (222) indicate Magnesium oxide MgO^23^. Characteristic peaks of calcium oxide CaO were characterized at the 2θ^o^ = of 32.25° (111), 54.04° (220), 63.89° (311), and 67.74° (222)^24^.

Ordinary Portland Cement (OPC) conforming to ASTM C150 Type was used in all concrete mixtures^25^. Chemical and Physical properties of cement are shown in Tables 1, 2, respectively.

**Table 1:** Chemical composition of the Portland cement and magnetite powder (wt%).

Material	Sio_2_	Al_2_O_3_	Fe_2_O_3_	Fe_3_O_4_	CaO	MgO	SO_3_	Na_2_O	K_2_O
Cement	20.2	5.0	2.6	-	64.1	1.58	2.5	0.2	0.7
Magnetite	0.80	0.28	-	97.6	0.20	-	-	0.06	0.02

**Table 2:** Physical properties of cement.

Characteristics	Value
Specific gravity	3.12
Initial setting time(min.)	48
Final Setting time(min.)	615
Standard consistency	37

Tap water, complying with ASTM C1602, served as the mixing water^26^. The fine aggregate (natural river sand) met the grading requirements of ASTM C33, while the coarse aggregate (crushed granite) had a nominal maximum size of 20 mm also conformed to ASTM C33 specifications^27^. Table 3 represents the physical properties of aggregate. Magnetite powder (Fe₃O₄) with a density of 4770 kg/m3 was used as a partial replacement of sand by weight. The powder was sieved through a No. 120 sieve (125 µm opening) according to ASTM E11 to ensure particle size compatibility with fine aggregate fractions^28^.

**Table 3:** Physical characteristics of sand and coarse aggregate.

Characteristics	Sand	Coarse aggregate
Specific gravity	2.67	2.7
Fineness	2.31	–
Moisture content %	3.7	0
Bulk density(kg/m^3^)	1570	1450
Voids %	41	39
Water absorption %	1.47	0.8

### Samples preparation and methods

#### Samples preparation and methods for the microwave shielding test

Five cement tiles with dimensions 20 × 30 × 1 cm were prepared with different magnetite sand ratios. The reference sample (M0) consisted of 437 g of cement, 218 g of water, and 874 g of sand. Samples 1 (M1), 2 (M2), 3 (M3), and 4 (M4) were prepared in the same manner except that 10, 20, 30%, and 40 wt% of sand were replaced by magnetite sand, respectively.

#### Test for EMW shielding

**Fig. 3:**
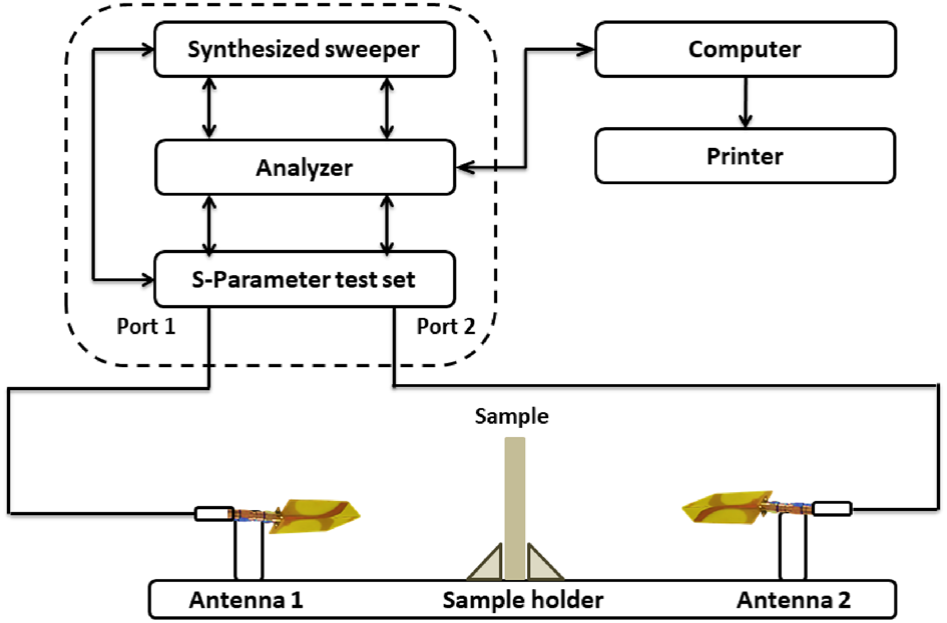
The free-space measurement system.

For the studies of electromagnetic shielding (EMS) response of Egyptian Black sand’s magnetite in the prepared cement-based tiles, a free-space measurement system that mainly consists of a pair of end-fire antennas, coaxial cables, and a Rohde & Schwarz ZVA67 vector network analyzer was used. The measurements were performed in the frequency band (2–12 GHz). The S21 parameter was measured for the five cement tiles M0, M1, M2, M3, and M4, as shown in Fig. 3.

#### Sample preparation for mechanical test

Five concrete mixes were prepared to evaluate the effect of magnetite powder as a sand replacement.

A reference concrete (RC) mix with 0% magnetite was used as a control. The experimental mixes, M10, M20, M30, and M40, incorporated 10, 20, 30, and 40% magnetite powder as a replacement for sand (by weight), respectively. Series containing magnetite powder were designated as M, while the digit next to the letter represents the amount of MP incorporated. All mixes had a constant water-to-cement ratio (w/c) of 0.50, and the total volume of aggregate was kept constant. The concrete mixtures were prepared in the following percentages: 1:1.5:3 cement, fine aggregate, and coarse aggregate, respectively, see Table 4. The magnetite replaced an equivalent weight of sand in each case. Dry materials were first mixed thoroughly, and water was added gradually during mixing. The mixing procedure followed ASTM C192^29^. Fresh concrete was placed into molds. The specimens were cast into 150 mm cubes for compressive strength testing, 150 × 300 mm cylinders for splitting tensile strength testing (ASTM C496), and 100 × 100 × 400 mm prisms for flexural strength testing (ASTM C78), and T 40 × 40x4 cm^3^ prisms for shielding tests^30,31^. After 24 h, samples were demolded and cured in a water bath at 27 ± 2 °C, in accordance with ASTM C511, until testing at 28 days^32^.

**Table 4:** Proposed mix proportion and slump test results.

Mix No	% of replacement	Cement kg/m^3^	Water kg/m^3^	F. Aggregate kg/m^3^	C. Aggregate kg/m^3^	Magnetite kg/m^3^	Slump mm
RC	0	450	225	675	1350	0	150
M10	10	450	225	607.5	1350	67.5	142
M20	20	450	225	540	1350	135	132
M30	30	450	225	472.5	1350	203	125
M40	40	450	225	405	1350	270	118

#### Test on fresh concrete

##### Workability

The workability of fresh concrete was evaluated using the slump test, in accordance with ASTM C143/C143M – Standard Test Method for Slump of Hydraulic-Cement Concrete^33^.

##### Tests on hardened concrete

###### Dry density

The dry density of the hardened concrete specimens was determined using the water displacement method, following the principles outlined in ASTM C642 – Standard Test Method for Density, Absorption, and Voids in Hardened Concrete^34^.

###### Compressive strength test

For the investigation of compressive strength, three 150 mm concrete cubes were prepared from each mixture with a measurement tolerance of ± 0.05 mm for their dimensions. The specimens were treated under normal conditions (as per ASTM 192) and tested for 28 days using a Universal Test Machine. The test results, which must be rounded to the nearest 0.1 N/mm, have an expanded uncertainty of 1.21 N/mm^2^ at the given level of confidence^29^.

###### Splitting tensile test

Three 150 × 300 mm cylinder specimens were prepared from each mixture to determine its tensile strength. The specimens were treated in water at room temperature for 28 days. Subsequently, they were tested for splitting tensile strength as per ASTM C496/C496M-17, and the average result was recorded^30^.

###### Flexural strength

Twelve 100 × 100 × 400 mm beam specimens were prepared for the flexural strength test. The beams were treated in water for 28 days. Then, three beams from each mixture were tested to failure by applying a center-point load, in accordance with ASTM C78/C78M-18. The average strength from these tests was then recorded for each case^31^.

## Results and discussion

### Electromagnetic wave shielding measurement

**Fig. 4:**
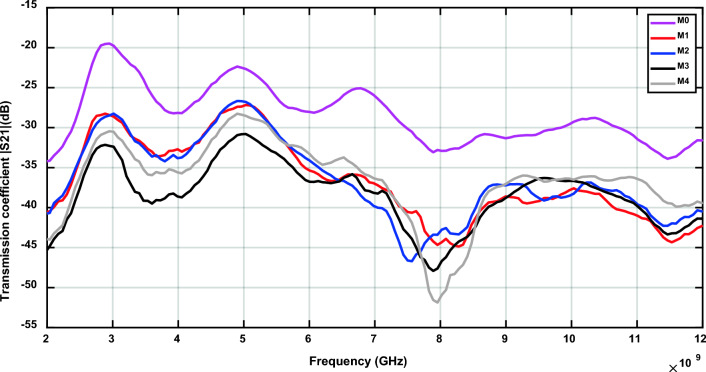
Transmission coefficients of the prepared samples with different concentrations of Egyptian Black sand’s magnetite.

**Fig. 5:**
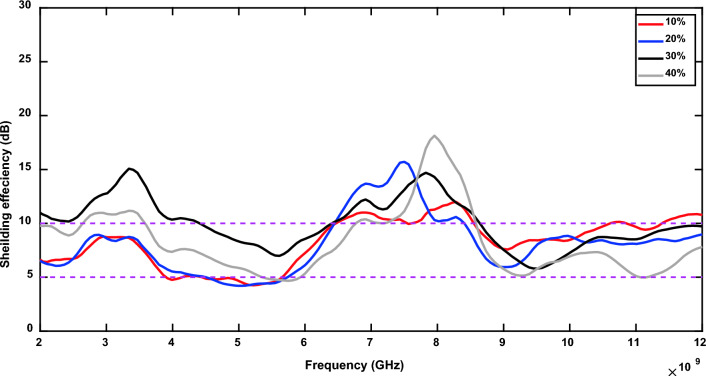
Electromagnetic shielding effectiveness of the prepared samples with different concentrations of Egyptian Black sand’s magnetite.

The measurement of S21 for the cement tile M0 (tile with 0% magnetite) is used as a reference to depict the degradation in electromagnetic waves caused by the increase of magnetite ratio in the cement tiles, as shown in Fig. 4. The four prepared cement tiles achieve moderate (> 3 dB) to high (> 10 dB) EMSE as shown in Fig. 5, where the shielding effectiveness is expressed in terms of attenuation of power in dB (Table 5).

**Table 5:** Effect of magnetite ratio on EMSE and optimal frequency range for samples from M1 to M4.

Samples	Magnetite ratio (%)	EMSE (dB)	frequency (GHz) optimal EMSE	frequency range (GHz) EMSE$\ge$10 dB (GHz)
M1	10	11.93	8.28	6.46 – 8.54
M2	20	15.70	7.56	6.46 – 8.41
M3	30	14.70	7.89	6.46 – 8.67
M4	40	18.14	7.95	6.78 – 8.60

Table 6 provides a comparative analysis of the different materials used for electromagnetic shielding in cement composites. Based on the relative advantages and disadvantages of using these materials, Y. Bai et al., and B. Li et al., study, which used reinforced carbon fibers to shield buildings from electromagnetic waves, showed results reaching a maximum value of 27.2 dB and 38.4 dB, respectively^16,35^. X. Zhang, et al., and N. Lee et al., used carbon nanotubes as a cement additive, which demonstrated a high reflection loss reached55, and 57.9 dB, respectively^36,37^. L. Gao et al. used silicon carbide (SiC) as a cement filler, demonstrating an insulation capacity of 16.25 dB.^38^. Z. Yang et al., added waste copper swarf to cement to shield buildings from electromagnetic waves, and the results showed a reflection loss of 4.5 dB.^11^.H. Li, M et al., and C. Ma et al., added Carbon Black, and the result of this addition was 29.67 dB ^39,40^. Finally, J. Guo et al., brought samples of cement and (Fe3S4)/reduced graphene oxide to reach a reflection loss result of 20 dB^41^. Tao Chen, et al. added carbon nanofiber reinforced and measured electromagnetic shielding effectiveness (SE_E_) of concrete. The result showed that the minimum of CNFs reinforced concrete is − 9.2 dB, and the maximum SE_E_ is—67.0 dB ^42^.

**Table 6:** Comparison between different materials for electromagnetic shielding and their performance.

Material	Shielding mechanism	Effectiveness	Cost	Dispersion in cement	Mechanical properties impact	Ref
Egyptian Black sand’s magnetite	Reflection, absorption	High	Very Low	Relatively Easy	Significant Improvement	Experimental test
Carbon Fibers	Reflection, absorption	Moderate	Low to Medium	Relatively Easy	Moderate Improvement	^16,35^
Carbon Nanotubes	Absorption, reflection	High	High	Challenging	Significant Improvement	^36,37^
silicon carbide (SiC)	Absorption, reflection	Low	low	Relatively Easy	Significant Improvement	^38^
waste copper swarf	Reflection, absorption	Very low	Very Low	Relatively Easy	–-	^11^
Carbon Black	Absorption, reflection	Moderate	Very Low	Relatively Easy	Significant Improvement	^39,40^
(Fe_3_S_4_)/reduced graphene oxide	Reflection	Moderate	Medium	Relatively Easy	––	^41^

### Workability

A consistent reduction in slump was observed with increasing magnetite content. The slump decreased from 150 mm in the reference mix (M0) to 118 mm in M40, indicating a 21% decrease in workability, see Table 4. The reduction in workability with magnetite is consistent with Ghazanlou et al.^43^, who observed a 20–25% decrease in slump with 40% magnetite substitution, attributing it to particle shape and density effects. Horszczaruk^44^ reported that magnetite reduced slump significantly when used in high percentages, especially in the absence of superplasticizers. in addition, Mydin et al.^45^ highlighted the need for water-reducing admixtures when magnetite is used to maintain target workability without increasing the water-cement ratio. The reduction in slump with increasing magnetite content can be attributed to several factors; ⁠Higher Specific Gravity and Particle Packing, magnetite has a much higher specific gravity (~ 5.2) compared to normal aggregates (~ 2.6–2.7). As magnetite content increases, the mix becomes heavier and denser, requiring more energy to flow. ⁠The Rough Surface Texture is a second reason, the relatively angular and rough texture of magnetite particles increases internal friction, thereby decreasing the ease of flow of fresh concrete. Finally, increased Surface Area, the inclusion of fine magnetite particles can increase the specific surface area, demanding more water for wetting, which reduces free water available for lubrication. Although workability decreased, the slump values remained within acceptable ranges for conventional placement methods. For applications requiring higher workability or flow, the use of superplasticizers or viscosity-modifying agents is recommended when magnetite is used. The trade-off between mechanical performance and workability must be managed carefully in practical applications.

### Dry density

A progressive increase in dry density was observed with higher magnetite content. The density rose from 2360 kg/m3 in the control mix (M0) to 2670 kg/m3 in the M40 mix, reflecting a 13.1% increase in mass per unit volume due to the incorporation of high-density magnetite particles. This increase is directly attributed to the high specific gravity of magnetite (~ 4.77), compared to natural sand (~ 2.65). The denser particles occupy the same volume but significantly increase the total mass, thus raising the dry density of the concrete. This is in agreement with Ghazanlou et al.^43^, who reported that partial replacement of fine aggregates with magnetite led to an increase in concrete density from ~ 2300 to ~ 2700 kg/m^3^, depending on the magnetite percentage. In another study, Ghazanlou and Ashraf^46^ observed that incorporating iron-based industrial wastes (like magnetite) into concrete mixes resulted in denser and more durable concrete, especially suitable for radiation shielding applications. Mydin et al.^45^ also highlighted that magnetite’s use improved the mechanical mass and unit weight of concrete, enabling its classification as heavyweight concrete when replacement ratios exceeded 30%

### Mechanical properties

#### Compressive strength

**Fig. 6:**
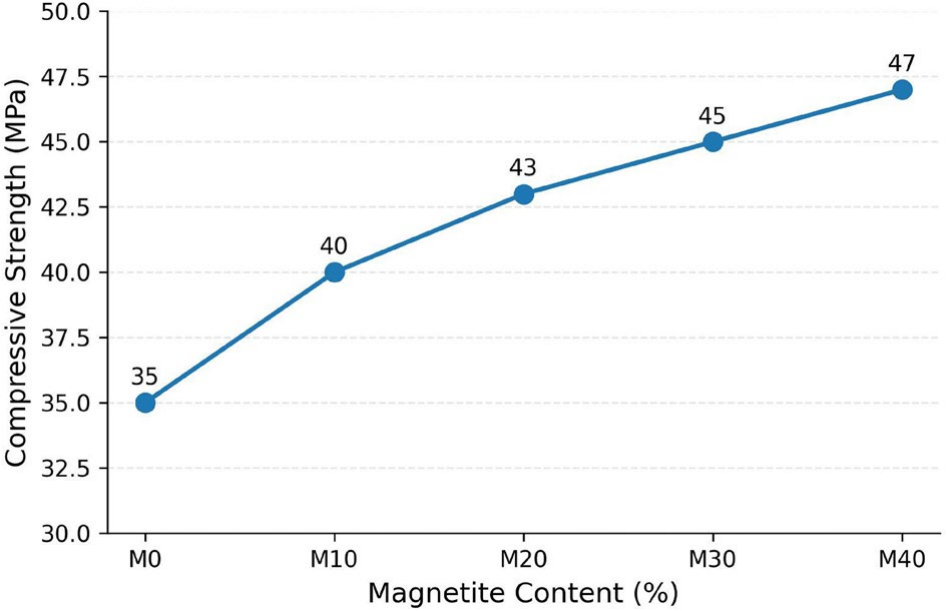
Effect of percentage of magnetite on compressive strength.

The compressive strength increased progressively from 35 MPa (M0) to 47.25 MPa (M40) (Fig. 6), representing an improvement of approximately 35%. This enhancement indicates that magnetite incorporation can significantly improve the load-bearing capacity of the concrete matrix when used at appropriate replacement levels. The strength improvement is primarily attributed to the high density and filler effect of magnetite, which enhances particle packing, reduces internal voids, and refines the pore structure, leading to a denser and more compact cementitious matrix. Similar strengthening mechanisms have been reported for cementitious composites incorporating Fe₃O₄ micro/nanoparticles, where improved packing density and pore refinement contributed to enhanced compressive performance^43^. In addition, a review by Horszczaruk^44^ highlighted that magnetite-based additions can improve cement matrix compactness and mechanical strength when the dosage is optimized. Although SEM analysis was not conducted in the present study, the compressive strength improvement is consistent with these established microstructure–property relationships. Therefore, future work will include SEM-based characterization to examine magnetite dispersion, pore refinement, and interfacial transition zone (ITZ) morphology to directly confirm the proposed strengthening mechanisms.

#### Tensile strength

**Fig. 7:**
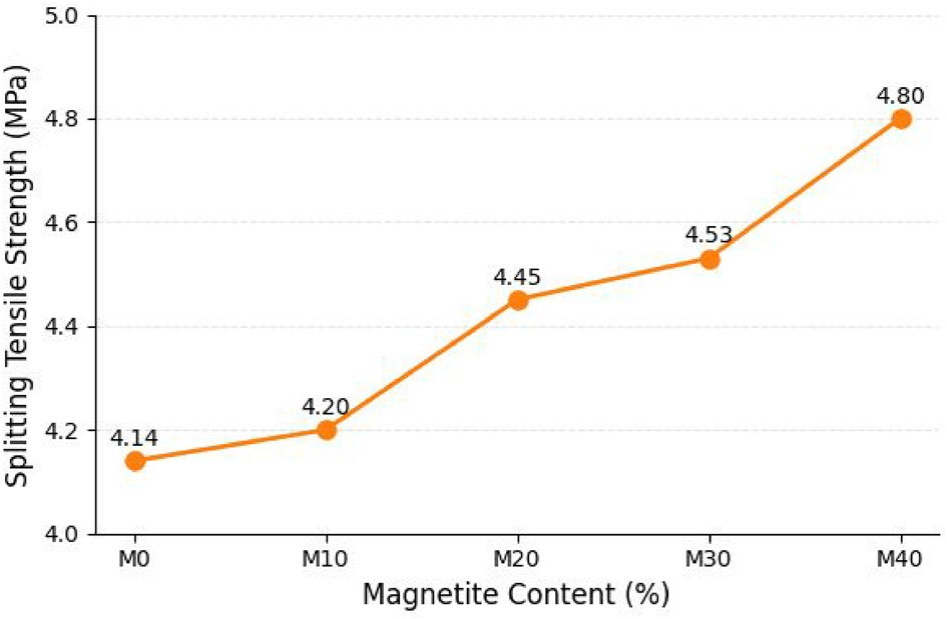
Effect of percentage of Magnetite on splitting tensile strength.

As shown in Fig. 7, the splitting tensile strength increased from 4.14 MPa (M0) to 4.80 MPa (M40), corresponding to an improvement of approximately 16%. The increase is smaller than that observed in compressive strength, indicating that while magnetite contributes to matrix densification, its influence on tensile load-carrying capacity remains relatively modest. This is expected because tensile behavior in concrete is strongly governed by crack initiation and propagation through weak zones such as pores and the interfacial transition zone. The observed tensile strength enhancement is consistent with improved crack resistance and delayed crack development noted in the fracture behavior of magnetite-containing mixes, which exhibited narrower cracks and less abrupt failure compared with M0. Similar observations have been reported by Mydin et al.^45^, who noted that improvements in matrix bonding can increase tensile-related performance; however, substantial tensile enhancement typically requires additional crack-control mechanisms such as fibers or polymer modification. The improvement observed here can therefore be reasonably attributed to reduced defect concentration and improved stress transfer due to the filler effect and enhanced matrix continuity.

#### Flexural strength

**Fig. 8:**
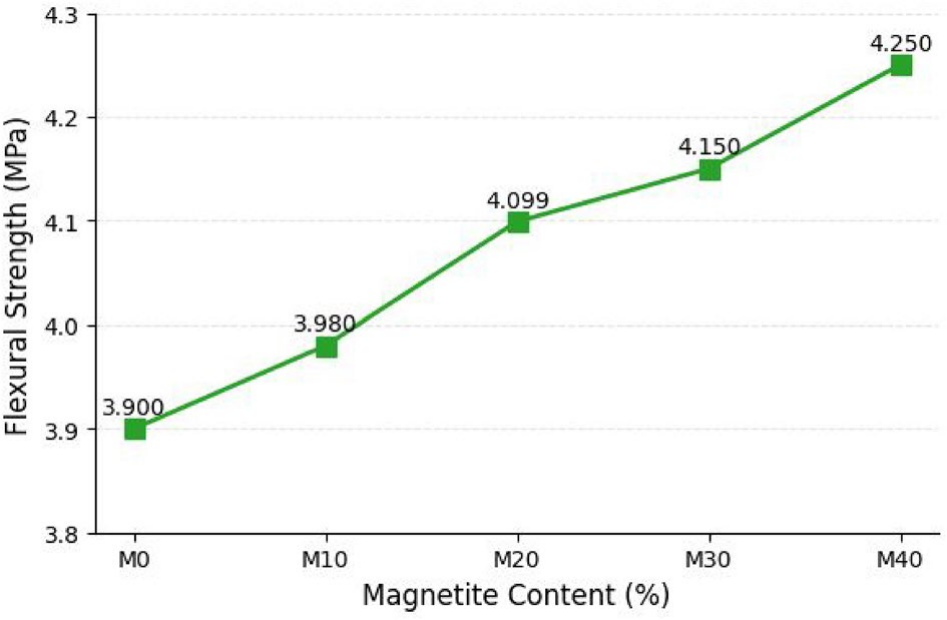
Effect of percentage of Magnetite on flexural strength.

The flexural strength increased from 3.90 MPa (M0) to 4.25 MPa (M40), representing an improvement of approximately 9% (Fig. 8). Although the increase is modest, it indicates improved resistance to bending-induced tensile stresses and enhanced microcrack control compared with the reference mix. This trend aligns with the fracture behavior observations, where magnetite-containing mixes (particularly M30 and M40) exhibited narrower crack widths and more tortuous crack paths, suggesting delayed crack propagation and improved energy dissipation relative to M0. The enhanced flexural performance is attributed to improved particle packing and matrix densification, which reduce micro-voids and stress concentration sites, thereby increasing resistance to crack initiation and propagation under bending. Similar effects have been discussed by Ghazanlou et al.^43^, who reported that magnetite-containing cementitious composites can exhibit improved flexural performance due to increased compactness and reduced micro-defects. In addition, studies on multifunctional cementitious composites have shown that tailored microstructures and functional fillers can enhance crack-growth resistance and improve fracture-related performance (Wang et al.^47^; Xiao et al.^48^). Although the present study does not quantify fracture toughness or fracture energy, the observed flexural improvement and fracture patterns support a transition toward more controlled, quasi-brittle failure in magnetite-modified mixes.

#### Fracture behavior

The fracture behavior of the concrete specimens was evaluated based on visual observation during mechanical testing and post-failure examination of the fractured surfaces. The reference concrete (M0) exhibited a predominantly brittle failure mode, characterized by sudden fracture and the formation of relatively wide and continuous cracks, which is typical of conventional concrete with limited energy dissipation capacity. In contrast, concrete mixes incorporating magnetite (particularly M30 and M40) demonstrated less abrupt failure behavior, with narrower crack widths, more tortuous crack paths, and comparatively more compact fractured surfaces. These observed fracture features indicate delayed crack initiation, reduced crack propagation rates, and improved energy dissipation, consistent with enhanced quasi-brittle behavior. Comparable trends have been reported in multifunctional cementitious composites that are designed to improve both mechanical performance and functional response; for example, Wang et al.^47^ observed that additions of fine reinforcing phases in multifunctional composites led to more gradual post-peak responses and enhanced crack resistance relative to plain matrices, attributing this to improved microstructural bridging and energy dissipation mechanisms. Similarly, Xiao et al.^48^ reported that cementitious composites engineered for combined mechanical robustness and electromagnetic wave absorption showed notably more tortuous crack paths and increased fracture resistance relative to conventional paste systems, consistent with enhanced structural continuity and microstructural refinement. The improved fracture response in the magnetite-modified concretes can be attributed to the densification of the cementitious matrix due to the filler effect of fine magnetite particles, which reduces internal voids and strengthens the interfacial transition zone between the aggregate and cement paste. The increased density and improved particle packing hinder crack coalescence and promote stress redistribution prior to failure. Mechanisms analogous to those observed in other multifunctional composites where tailored microstructures and distributed functional fillers impede crack propagation and enhance energy dissipation.

## Economic value

It is clear from the current study and from comparison with some studies that focused on shielding from electromagnetic waves in the frequency range from 2 to 12 GHz that the materials that achieved a high degree of protection are materials manufactured using high technologies and therefore very expensive, such as Carbon Fibers, Carbon Nanotubes, and (Fe_3_S_4_)/reduced graphene oxide. They are also difficult to obtain in large quantities that are suitable for building dense buildings. On the other hand, materials that are easy to obtain in larger quantities, such as waste copper swarf and Carbon Black, do not achieve a significant result in reducing the values of electromagnetic waves passing through concrete. However, in our study, magnetite sand achieved a high degree of shielding from electromagnetic waves, in addition to improving the mechanical properties of concrete, at a very low cost, as the price of a ton of Egyptian magnetite sand was recorded at $300 per ton, according to the pricing of the Egyptian Black Sand Company.

## Conclusion

Electromagnetic waves are a common means of communication used in many aspects of life, but they also have harmful effects. Therefore, the need to reduce electromagnetic pollution has emerged. So, a study has been conducted to reduce this pollution using environmentally available materials in building components. The study shows that increasing the percentage of magnetite sand from 10 to 40% as a partial replacement for sand gradually improves the effectiveness of electromagnetic shielding. Specifically, the M40 sample, which was replaced by 40% magnetite, achieved an optimal electromagnetic shielding effectiveness of 18.14 dB at a frequency of 7.955 GHz. This high attenuation is attributed to magnetite’s magnetic and conductive properties, which facilitate the absorption and reflection of electromagnetic waves.

The incorporation of magnetite also positively affects the mechanical performance of concrete mixes. The higher magnetite percentage resulted in a significant increase in dry density (up to 13.1% in M40) due to its high specific gravity. This densification, combined with the magnetite particle filler effect, resulted in significant improvements in compressive strength (up to 35% for M40), splitting tensile strength (up to 16%), and flexural strength (up to 9%). While workability decreased with increasing magnetite content, shrinkage values remained within an acceptable range. Overall, magnetite from Egyptian black sand represents a promising, cost-effective, and dual-use solution for producing building materials that not only provide effective electromagnetic shielding but also exhibit superior mechanical properties.

## Data Availability

The datasets used and/or analysed during the current study available from the corresponding author on reasonable request.
